# Activation of Nicotinic Cholinergic Receptors Prevents Ventilator-Induced Lung Injury in Rats

**DOI:** 10.1371/journal.pone.0022386

**Published:** 2011-08-08

**Authors:** Fabienne Brégeon, Francois Xeridat, Nicolas Andreotti, Hubert Lepidi, Stéphane Delpierre, Antoine Roch, Sylvie Ravailhe, Yves Jammes, Jean-Guillaume Steinberg

**Affiliations:** 1 UMR MD2 P2COE, Institut Fédératif de Recherche Jean-Roche, Faculté de Médecine, Université de la Méditerranée Aix-Marseille II and Explorations Fonctionnelles Respiratoires de l’ Hôpital Nord, Assistance Publique – Hôpitaux de Marseille, Marseille, France; 2 Unité de Recherche sur les Maladies Infectieuses et Tropicales (URMITE), Faculté de Médecine, Université de la Méditerranée and Réanimation Médicale and Service des Détresses Respiratoires et Infections Sévères de l’Hôpital Nord, Assistance Publique – Hôpitaux de Marseille, Marseille, France; 3 ERT 62, ‘Ingénierie des Peptides à visée Thérapeutique’, Institut Fédératif de Recherche Jean-Roche, Université de la Méditerranée Aix-Marseille II, Marseille, France; 4 Unité des Rickettsies, URMITE CNRS-IRD 198 UMR 6236, and Service D'anatomie Pathologique et Neuropathologie de l’ Hôpital Timone, Assistance Publique – Hôpitaux de Marseille, Marseille, France; McMaster University, Canada

## Abstract

Respiratory distress syndrome is responsible for 40 to 60 percent mortality. An over mortality of about 10 percent could result from additional lung injury and inflammation due to the life-support mechanical ventilation, which stretches the lung. It has been recently demonstrated, in vitro, that pharmacological activation of the alpha 7 nicotinic receptors (α7-nAChR) could down regulate intracellular mediators involved in lung cell inflammatory response to stretch. Our aim was to test in vivo the protective effect of the pharmacological activation of the α7-nAChR against ventilator-induced lung injury (VILI). Anesthetized rats were ventilated for two hours with a high stretch ventilation mode delivering a stroke volume large enough to generate 25-cmH_2_O airway pressure, and randomly assigned to four groups: pretreated with parenteral injection of saline or specific agonist of the α7-nAChR (PNU-282987), or submitted to bilateral vagus nerve electrostimulation while pre-treated or not with the α7-nAChR antagonist methyllycaconitine (MLA). Controls ventilated with a conventional stroke volume of 10 mL/kg gave reference data. Physiological indices (compliance of the respiratory system, lung weight, blood oxygenation, arterial blood pressure) and lung contents of inflammatory mediators (IL-6 measured by ELISA, substance P assessed using HPLC) were severely impaired after two hours of high stretch ventilation (sham group). Vagal stimulation was able to maintain the respiratory parameters close to those obtained in Controls and reduced lung inflammation except when associated to nicotinic receptor blockade (MLA), suggesting the involvement of α7-nAChR in vagally-mediated protection against VILI. Pharmacological pre-treatment with PNU-282987 strongly decreased lung injury and lung IL-6 and substance P contents, and nearly abolished the increase in plasmatic IL-6 levels. Pathological examination of the lungs confirmed the physiological differences observed between the groups. In conclusion, these data suggest that the stimulation of α7-nAChR is able to attenuate VILI in rats.

## Introduction

Mechanical ventilation is the main life-sustaining tool in Acute Respiratory Distress Syndrome (ARDS), but even low tidal volume strategies may cause the undesirable side-effects of cyclic hyper-inflation of some lung areas [1]. Hyper-inflation of these areas exposes the lung to ventilator-induced lung injury (VILI) which is characterized by increased endothelial and epithelial permeability and inflammatory processes [2]. Therefore, protection against VILI appears essential.

Inflammatory processes involving lung cytokines could play a major role in VILI. One of the systems regulating cytokine release is the alpha7 nicotinic acetylcholine receptors (α7-nAChR). These receptors are expressed on macrophages and epithelial cells [3] and can be activated by acetylcholine released from efferent vagal endings. The anti-inflammatory effect of the vagally-mediated cholinergic pathway has been tested either in septic and non septic models [4]–[12]. Fewer and contrasting data have been published about lung inflammation [3], [5], [13]. In endotoxinic rats, Bernik et al. have reported that vagal electrical stimulation was unable to reduce pulmonary tumor necrosis factor synthesis whereas a significant reduction was observed in serum and myocardium [5]. By contrast, data from Su et al. in mice exposed to acid-induced acute lung injury or to Escherichia coli-induced lung injury strongly suggest an anti-inflammatory effect of the vagally-mediated cholinergic pathway at the lung level [3], [13]. As for VILI, we found only one in vivo report which supposes the existence of an interaction between stretch-induced lung cytokine release (20 mL/kg tidal volume) and the vagal anti-inflammatory pathway [14]. In this above mentioned report, additional in vitro experiments have also shown that specific agonist or antagonist of the α7-nAChR acts on the intracellular mediators involved in cell inflammatory response to stretch such as pJNK (pro-inflammatory) and Fas, Daxx, pJNK and Bad (pro-apoptotic) [14]. However, the protective effect of α7-nAChR activation against VILI still remains to be assessed in vivo.

The aim of the present study was to assess, in a rat model of single-hit VILI, whether a pre-treatment with an agonist of the α7-nAChR could attenuate the ventilator-induced impairment in lung function. Furthermore, we examined whether improvement, if any, was associated to a lower inflammatory response. We exposed a group of rats to high stretch mechanical ventilation (HV) at a stroke volume large enough to generate 25-cmH_2_O airway pressure. In other groups, the same ventilation setting was applied either during vagal electrostimulation or after pre-treatment with the specific agonist of the α7-nAChR, PNU-282987. The airway pressure and arterial blood gases were measured during the experiments. The respiratory system compliance was measured at the end, completed by further lung pathological examination and lung IL-6 and substance P assays. Our data show that the stimulation of the α7-nAChR was able to prevent lung impairment in this animal model of VILI.

## Methods

### Animals

The experiments and protocols we used were in accordance with the European law and its French version laid out in statutory requirements for alive animal experiments (*articles R214-87* to *R215-10* of *Code Rural*, law #*76-629* from July 10^th^, 1976*/*law *#2001-464* from May 29^th^, 2001 (published in *JORF* on May 31^st^, 2001). Conforming to these laws, the experiments were always performed under direct control of holders of a name-specific authorization delivered by Préfecture-des-Bouches-du-Rhone administration (authorization numbers #13-46 for SD and #13-437 for AR). All the interventions on animals conforming to the guidelines laid out in the Guide for the Care and Use of Laboratory Animals, were performed according to the requirements of the ethic committee of the Institut-Fédératif-de-Recherche-Jean-Roche, Université-de-la-Méditerranée, and were made in its rooms (institution permit number: #C13-055-8, delivered by Préfecture-des-Bouches-du-Rhone-Direction-Départementale-des-Services-Vétérinaires on January 30^th^, 2007). According to the *directive #2010/63/UE of the PARLEMENT EUROPÉEN* and of the *CONSEIL EUROPEEN* published on September 22^nd^, 2010, about the protection of animals used for scientific aims, no additional specific agreement number was required for this study in non decerebrated rats, using euthanasia under anesthesia plus analgesia for which animals were never awakened in the course of the experiment.

Adult Sprague-Dawley male rats (n = 49, mean body weight, BW = 427±10 g) were anaesthetized with an intraperitoneal mixture of sodium pentobarbitone (20 mg/kg) and ethyl-carbamate (0.5 g/kg). After tracheotomy, the left carotid artery was catheterized for arterial blood pressure measurement (ABP) and blood samplings (electromanometer Statham P23 Db, Puerto Rico, Puerto Rico). An external jugular vein was cannulated for drug injections. In every animal, both cervical vagus nerves were dissected and exposed for further electro-stimulation or not.

To maximize lung injury following mechanical ventilation, rats were exposed to HV by adjusting the stroke volume so that the airway pressure (Paw) reached initially 25 cmH_2_O (HV_25_ groups). These animals were assigned to four groups and were ventilated for a maximum of two hours:

- One group was pre-treated with saline twenty minutes before starting HV (one intra-peritoneal injection of 2.4 ml/kg plus one intravenous injection of 1 mL/kg: HV_25_-sham group, n = 7). This group constituted the reference for VILI.
- One group had electrical stimulation of the cervical vagus nerves before and during HV (HV_25_-stim group, n = 7) in order to confirm the efficiency of vagally-mediated protection against this model of VILI.
- One group was pre-treated with the selective antagonist methyllycaconitine (MLA) by a single intravenous injection performed ten minutes before starting HV ventilation and electrical stimulation of the vagus nerves (HV_25_-stim/MLA group, n = 7). This group aimed to examine if vagally-mediated protection against VILI involved the α7-nAChR.
- One group was pre-treated with the selective α7-nAChR agonist PNU-282987 injected intraperitoneally (2.4 mg/kg) twenty minutes before starting high pressure ventilation (HV_25_-PNU group, n = 7). This aimed at testing whether the activation of the α7-nAChR was protective independently of vagal activation.

**Figure 1:**
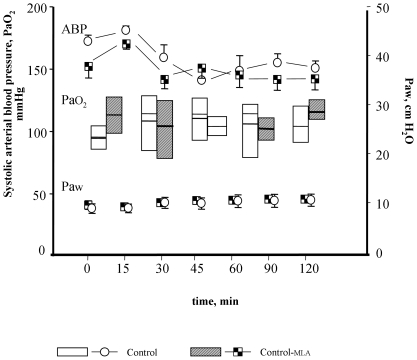
Time course of arterial oxygen partial pressure (PaO_2_), airway pressure (Paw), and systolic arterial blood pressure (ABP) in rats ventilated with conventional ventilation and pre-treated with saline (Control) or methyllycaconitine (Control-MLA). Data are presented as mean + SEM for ABP (lines and plots) and Paw (plots). PaO_2_ is presented as box plots: the box represents the 25 and 75 percentiles, mean and median are presented as horizontal lines. No significant change was measurable.

One group of rats ventilated for two hours with conventional ventilation (10 mL/kg stroke volume, 7–8 cmH_2_O Paw and positive end expiratory pressure at 1 cmH_2_O) was used for reference data (Control group, n = 7). Their main data are reported in figure 1 and Table 1. The toxicity of MLA has been related to neurological disorders resulting in tonic convulsions and seizures followed by respiratory paralysis [15] but we have found no report on its direct cardio-respiratory toxicity in literature. However, we tested the possibility of a cardio-respiratory toxicity of this molecule during ventilatory support in our model. Two additional groups were performed, one receiving conventional ventilation (Control-MLA group, n = 7), another one receiving HV (HV_25_-MLA group, n = 7). In the Control-MLA group, minor circulatory or respiratory changes were observed, similar to that observed in Controls (Figure 1). In the HV_25_-MLA group, a cardio-respiratory impairment occurred but did not significantly differ from that of the HV_25_-sham group, showing a progressive decrease in ABP and increase in Paw (see Figure S1 A). At the end of the ventilation period, lung mechanics characteristics and lung weight indexed to the body weight did not significantly differ between both groups (see Figure S1 B, C, D).

**Table 1:** Variables measured after mechanical ventilation with conventional ventilation (controls) or after high stretch ventilation.

groups	Final PaO_2_, mmHg	Lung weight/body weight, g/kg	Crsi/BW, mL/cmH_2_O/kg
**Controls**	114±4	2.10 [1.94–2.21]	1.60±0.19
**HV_25_-sham**	66±7	5.10 [4.21–5.90]	0.35±0.11
**HV_25_-PNU**	90±2**	2.21 [2.00–2.30]***	1.67±0.14***
**HV_25_-stim**	88±5*	2.53 [2.18–3.14]***	1.29±0.12***
**HV_25_-stim/MLA**(at 45 minutes)	21±3***	6.29 [2.72–8.68]	0.16±0.02

Crsi/BW: static compliance of the respiratory system measured on inflation and indexed to the body weight. **HV_25_-sham**: injurious ventilation and saline injection. **HV_25_-stim**: injurious ventilation associated with bilateral electro-stimulation of the cervical vagus nerves. **HV_25_-stim/MLA:** injurious ventilation associated with bilateral electro-stimulation of the cervical vagus nerves and pre-treatment with alpha7 nicotinic acetylcholine receptor blocking agent MLA. **HV_25_-PNU** : injurious ventilation associated with pre-treatment with alpha7 nicotinic acetylcholine receptor agonist PNU.

*, ** and ***: respectively p<0.05, <0.01 and <0.001 versus HV_25_-sham.

### Chemicals

The α7-nAChR agonist was the PNU-282987 (Tocris Bioscience Ellisville, MO, USA), chosen for its selectivity [16]. It was injected intraperitoneally at a dose of 2.4 mg/kg, previously reported to be effective against lung inflammation in rodents [3]. The nicotinic receptor blocking agent methyllycaconitine (MLA), more specifically the 3-methyl-2,5-dioxopyrrole (Sigma-Aldrich Chemie. L’Isle d’Abeau Chesnes, St Quentin Fallavier France) was also chosen for its selectivity [17] and was injected intravenously at the previously reported single dose of 1 mg/kg in rodents [18].

### General procedure for injurious ventilation and electrical stimulation

Ventilation was delivered via a small animal volume-controlled ventilator (Harvard Rodent Ventilator Model 683). It was initially started with a conventional stroke volume to generate 7–8 cmH_2_O Paw for the first ten minutes. Then, high stretch ventilation was achieved by increasing the stroke volume so that Paw reached 25 cmH_2_O and was left unchanged until the end of the experiment. A 1-cmH_2_O end-expiratory pressure was applied. The respiratory rate was fixed at 50 b/min. The pump delivered room air enriched with CO_2_ via a low-flow flowmeter (Rota Oeflingen flowmeter, Baden, Germany) to avoid hypocapnia. The CO_2_ flow was adjusted to the arterial carbon dioxide partial pressure (PaCO_2_) measurement and ranged between 10 and 50 mL/min whereas the minute ventilation of the animals was approximately 500 mL/min. We controlled that the resulting inspired oxygen fraction was not hypoxic: it ranged between 20 and 20.5 % (Rapidox 1100® gas analyzer, Cambridge Sensotec Ltd, Cambs, England). In the HV_25_-stim and HV_25_-stim/MLA groups, both cervical vagus nerves were placed on bipolar platinum electrodes connected via an isolation unit to a Grass S8800 stimulator. The characteristics of the square pulses were: voltage = 5–10 V, duration = 2 ms, frequency = 5–10 Hz and were chosen according to previously published protocols [5], [10]. Vagal stimulation started 10 min before the beginning of mechanical ventilation and went on for the first ten minutes of non injurious ventilation. Thereafter, vagal stimulation was performed repeatedly for 10 min-periods with stimulation alternating with 10-min periods without stimulation, to avoid nerve damage by prolonged electrical stimulation. The effectiveness of the vagal stimulation was attested by the characteristic transient bradycardia and hypotension, named ‘vagal escape’ [19] (see Figure S2). The vagally-induced bronchospasm was observed when ventilation was not yet injurious (see Figure S3). When the 25 cmH_2_O Paw level had been reached, no further increase in Paw could be detected during vagal stimulation, probably because of the strong distension of airways and of an overlap of pressure levels (as illustrated in Figure S2).

#### Continuous monitoring

As indexes of lung function impairment, a decrease in blood oxygenation was expected on repeated arterial blood gas analyses (Radiometer ABL 330, Copenhagen) and a Paw increase was checked on the continuous monitoring (Gould TA 4000, Ballinviliers, France). A cardiovascular monitoring was also continuously performed.

#### Lung mechanics assessment

The rats were euthanized after two hours of ventilation by an intravenous overdose of 5 % sodium pentobarbitone or died spontaneously. Post-mortem volume-pressure relationships were determined for further calculation of the inspiratory compliance of the respiratory system (Crsi) indexed to the body weight (Crsi/BW). Then, the trachea was clamped at end-inspiration (inflated at 8 mL/kg) before sternotomy. The gross vessels were cut and blood passively emptied. The left lung was filled with 10% buffered formalin for histological analysis. The right lung was weighed and divided into three fragments that were frozen for further biochemical assays.

#### Histological analysis

Fixed lungs were divided in three equal parts from the apex to the bottom, defined as cranial, middle and caudal. Slices of three micrometers were made in the median part of each section, including the whole circumference of the lung. Two pathologists blinded to the group assignment examined the slides. As previously described [20], they quantified the five following histological criteria: edema, interstitial inflammatory cell infiltration, intra-alveolar inflammatory cell accumulation, hemorrhage and hyaline membranes. Their respective intensity was scored as zero (absent), + (mild), ++ (moderate), +++ (severe) and ++++ (very severe). The intensities obtained on each of the three slides were summed up (maximum = 12). A total composite score was calculated by adding the score of each representative criterion.

#### Enzyme-Linked Immunosorbent Assay Measurements

The IL-6 levels were measured in the supernatant of homogenized lung extracts and in plasma with sensitive Enzyme-Linked ImmunoSorbent Assay – ELISA - kits (ER2IL6, supplied by Thermo Fisher Scientific, Perbio Science France SAS, Brebières, France) and using the StatFax 3200 microplate reader (Awareness Technology Inc., Palm City, FL, USA).

#### Substance P (SP) extraction and detection

The tachykinin SP, possibly from neuronal and non neuronal origin in rat lungs [21], acts as a pro-inflammatory mediator and was previously reported to increase with the level of lung stretch and injury [22]. We used this molecule as a complementary marker of lung inflammation in our model. Detection and quantification were performed using high-performance liquid chromatography (HPLC, Onyx) on lyophilized lung extracts (1 mg per run) as previously described [22]. The area under the SP peak chromatogram was expressed in arbitrary units and used for comparisons. Scattering of data measured by sd coefficient corresponds to that of repeated measurements in the same aliquot collected from all individuals in each group.

### Statistics

The SigmaStat 3.0 program (Sigma Company, SPSS Inc., Erkrath, Germany) was used. Data were tested for normal distribution with the Kolmogorov-Smirnov test. When data distribution of continuous variables was not normal, median and quartile values were given in the text and individual data or box plots (median and quartile values) were presented in the figures. When data were normally distributed, we used mean ± SEM in the text and figures.

For most of the temporally repeated data, the changes over time were assessed in each group using a mixed-design analysis of variance model for repeated measures (mixed-design ANOVA model including between-subjects variable and random effect). In case of significant difference, Holm-Sidak comparison versus the first time with high pressure ventilation was performed. Multiple comparisons between groups were performed, using two-way ANOVA (treatment, individuals) or Mann-Whitney’s rank sum test when appropriate. They were followed by Holm-Sidak comparison versus HV_25_-sham group in animals that survived for the 2-h period of the experiment, except for the HV_25_-stim/MLA group in which the animal died prematurely. A p value ≤0.05 was used to determine statistical significance.

## Results

### Ventilator-induced lung injury

**Figure 2:**
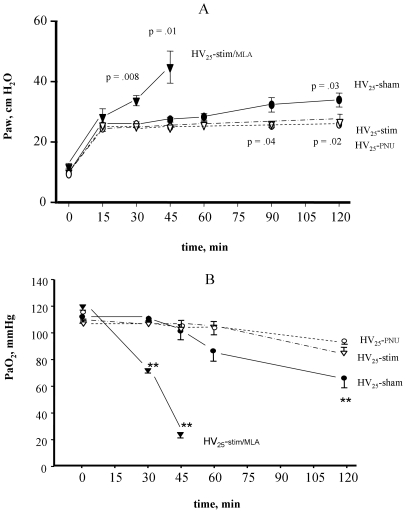
Time course of airway pressure (Paw, panel A) and arterial oxygen partial pressure (PaO_2,_ panel B), in animals exposed to high stretch ventilation. HV_25_-sham: animals exposed to 25 cmH_2_O Paw only. HV_25_-stim: animals exposed to 25 cmH_2_O Paw and receiving vagal stimulation. HV_25_-stim/MLA, vagal stimulation was associated to a pre-treatment with a specific antagonist of the alpha7 nicotinic acetylcholine receptor, methyllycaconitine (MLA). HV_25_-PNU: animals exposed to 25 cmH_2_O Paw and pre-treated with the alpha7 nicotinic acetylcholine receptor agonist PNU. Levels of significance versus HV_25_-sham are indicated on the graphs or by asterisks: ** p<0.01.

**Figure 3:**
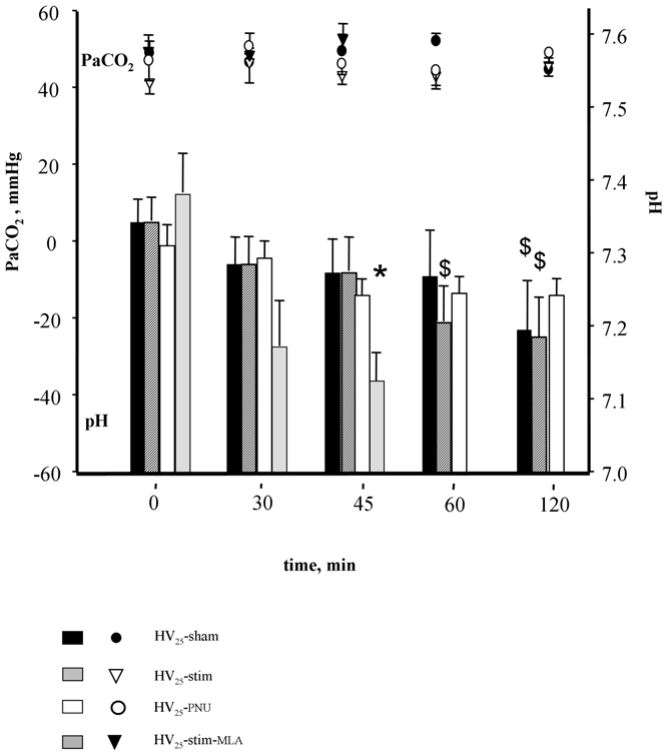
Comparative arterial blood carbon dioxide partial pressure (PaCO_2_, plots) and arterial pH (bars) in groups exposed to high stretch ventilation. HV_25_-sham: animals exposed to 25 cmH_2_O Paw only. HV_25_-stim: animals exposed to 25 cmH_2_O Paw and receiving vagal stimulation. HV_25_-stim/MLA, vagal stimulation was associated to a pre-treatment with a specific antagonist of the alpha7 nicotinic acetylcholine receptor, methyllycaconitine (MLA). HV_25_-PNU: animals exposed to 25 cmH_2_O Paw and pre-treated with the alpha7 nicotinic acetylcholine receptor agonist PNU. Symbol $ indicates significant differences versus baseline in a same group. Symbol * indicates significant differences versus HV_25_-sham group at the same time.

In animals exposed to HV, the average tidal volume indexed to the body weight was 24.33±0.21 mL/kg (corresponding to 10.30±0.20 mL), with no significant difference between groups. For conventional ventilation, the average tidal volume indexed to the body weight was 10.25±0.02 mL/kg (corresponding to 4.27±0.01 mL). At the fixed stroke volume, the HV_25_-sham group showed a progressive increase in Paw with time (figure 2 panel A) and a decrease in blood oxygenation (figure 2 panel B) while the PaCO_2_ was maintained in the normal range for all groups throughout the study period (figure 3).

**Figure 4:**
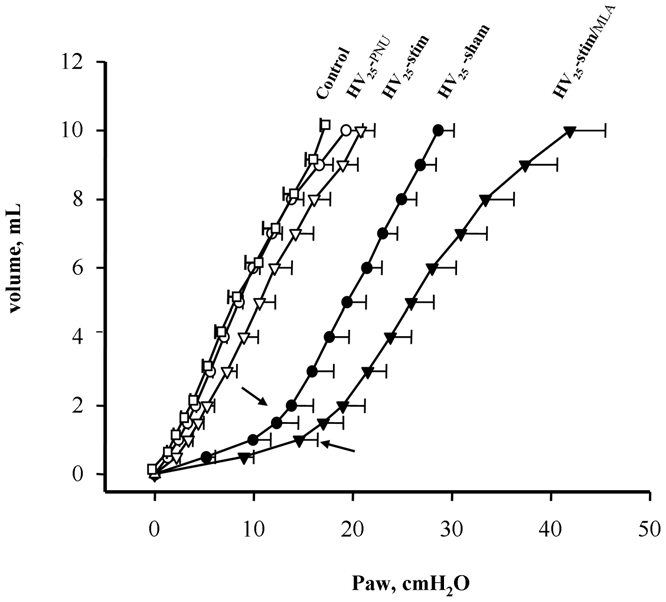
Respiratory system compliance. The pressure-volume curves during inflation in rats exposed to high stretch ventilation show the rightward shift and a lower inflection point (arrows) in unprotected groups. The reference curve from animals ventilated with non-injurious ventilation for two hours is also shown (Control). **HV_25_-PNU**: pre-treated with alpha7 nicotinic acetylcholine receptors agonist PNU (empty circles). **HV_25_-stim**: associated with vagal electrostimulation (empty reversed triangles). **HV_25_-sham**: pre-treated with saline (plain circles). **HV_25_-stim/MLA**: associated with vagal electrostimulation and pre-treatment with alpha7 nicotinic acetylcholine receptor blocking agent MLA (plain reversed triangles)

At the end of the experiments, HV_25_-sham animals had higher lung weight and lower respiratory system compliance than what was measured in the controls (Table 1). HV_25_-stim animals had significantly better lung mechanical properties than the HV_25_-sham ones (no measurable alteration of Crsi/BW and absence of increased Paw from the beginning of injurious ventilation), better gas exchange and lower lung weight (Table 1 and figure 2). Figure 4 shows that the volume-pressure relationship of the non-protected group (HV_25_-sham) shifted to the right, attesting of impaired lung mechanical properties, while it was close to that of controls in the HV_25_-stim group.

On the contrary, lung damage worsened in the HV_25_-stim/MLA group (Table 1, figure 2) and the shift to the right of the volume-pressure relationship of this group was even more pronounced than that observed in the HV_25_-sham group (figure 4).

Animals receiving direct α7-nAChR activation (HV_25_-PNU group) did not develop any significant physiological change in lung function (figure 2, Table 1). In a similar way to the HV_25_-stim group, the volume-pressure relationship of the HV_25_-PNU group did not clearly shift to the right (figure 4).

### Hemodynamics

**Figure 5:**
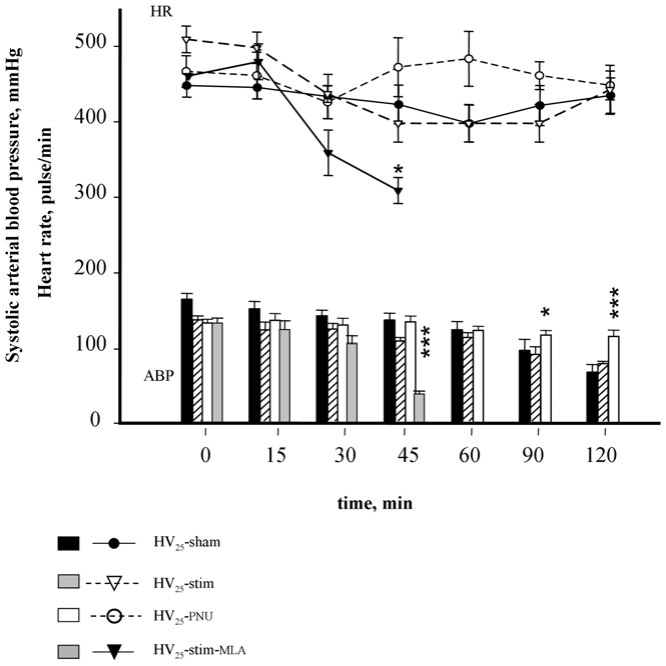
Time course of cardio-circulatory variables in animals exposed to high stretch ventilation. Heart rate (HR) and systolic arterial blood pressure (ABP) in animals ventilated at 25 cmH_2_O airway pressure only (HV_25_-sham), or associated with vagal stimulation (HV_25_-stim) or a specific antagonist of the alpha7 nicotinic acetylcholine receptor, methyllycaconitine (MLA) (HV_25_-stim/MLA), or without vagal stimulation but pre-treatment with the alpha7 nicotinic acetylcholine receptor agonist PNU (HV_25_-PNU). *p<0.05, ** p<0.01; *** p<0.001 versus HV_25_-sham.

A decrease in arterial pH was observed in all HV_25_ animals (figure 3). It reached statistical significance at the 120 min-period in the HV_25_-sham and the HV_25_-stim groups, did not reach statistical significance in the HV_25_-PNU group and was more pronounced and appeared earlier in the HV_25_-stim/MLA group than in the other groups. Concordantly, all groups but the HV_25_-PNU group had a progressive fall in systolic ABP (figure 5). Compared to HV_25_-sham rats, the HV_25_-stim animals tended to have a less pronounced fall but the difference did not reach statistical significance (end, systolic ABP = 89±3 mmHg in the HV_25_-stim group and 69±9 mmHg in the HV_25_-sham group). The outcome was particularly impaired in the HV_25_-stim/MLA group, (final systolic blood pressure = 46±5 mmHg (figure 5), associated to bradycardia, and premature deaths occurred in a context of cardio-respiratory failure (figure 5 and table 1). Inversely, systolic ABP remained above 100 mmHg in the HV_25_- PNU group, contrary to that observed in HV_25_- sham animals (at the end, systolic ABP = 134±10 mmHg in the HV_25_- PNU group, p<0.01 versus HV_25_-sham group).

### Lung inflammation

**Figure 6:**
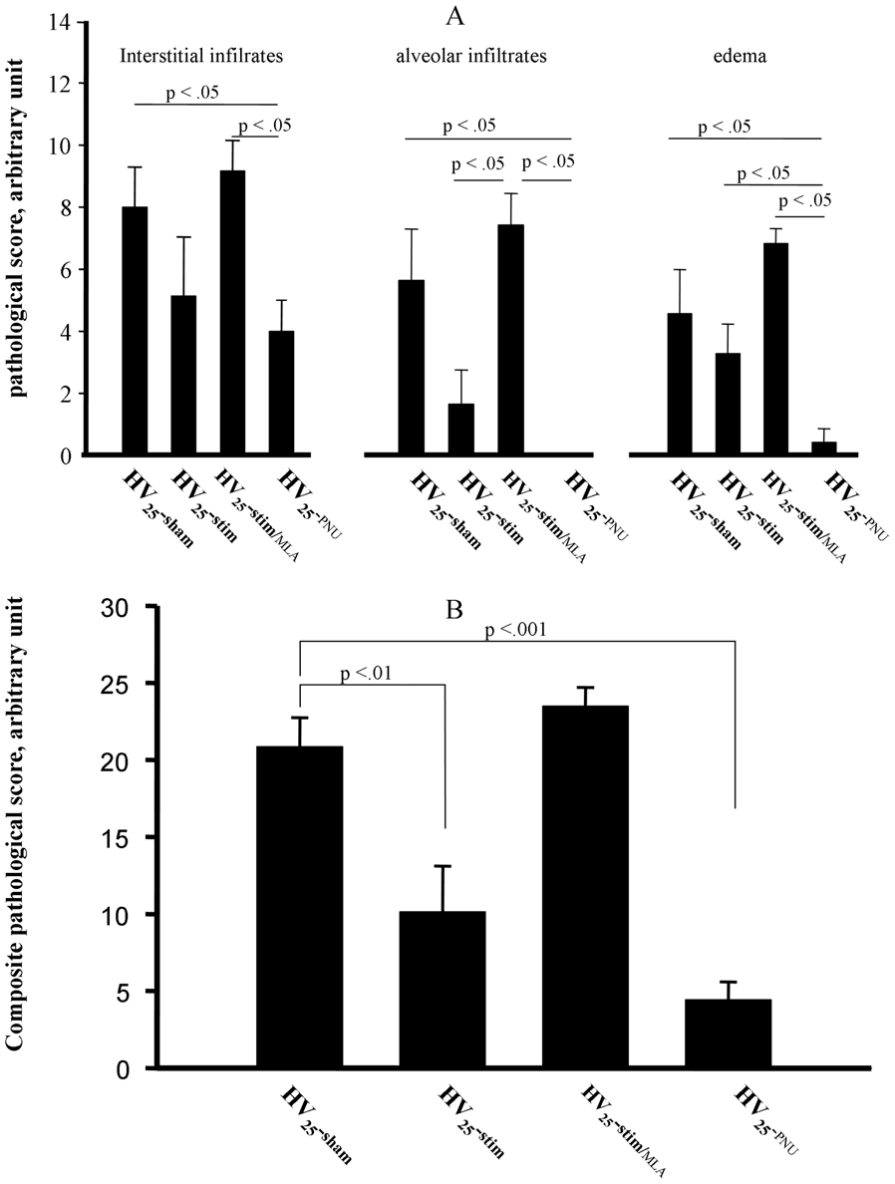
Lung injury scores in rats exposed to high stretch ventilation. Panel A represents individual score, panel B represents composite score. HV_25_-sham: injurious ventilation and saline injection; **HV_25_-PNU**: pre-treated with alpha7 nicotinic acetylcholine receptors agonist PNU; **HV_25_-stim**: associated with vagal stimulation; **HV_25_-stim/MLA**: associated with vagal stimulation and pre-treatment with alpha7 nicotinic acetylcholine receptor blocking agent MLA. Data are expressed as mean ± SEM. Levels of significance are indicated on the graph.

Pathological examination showed that inflammatory cell infiltration and edema caused by injurious ventilation (HV_25_-sham group) were present but minimized in the HV_25_-PNU group, weakly but significantly more pronounced in the HV_25_-stim group and increased in the HV_25_-stim/MLA group. The criteria of inflammation were the most discriminating. Only few cases of hemorrhage were observed: they concerned only the HV_25_-sham and HV_25_-stim/MLA groups. Hyaline membranes were absent. Edema, intra-alveolar cell accumulation and interstitial inflammatory cell infiltrates were the three selected criteria for the total composite score (sum of the two obtained intensities, minimum  = 0, maximum  = 36), which was significantly lower in the HV_25_-stim and HV_25_-PNU groups (figure 6 panels A and B).

**Figure 7:**
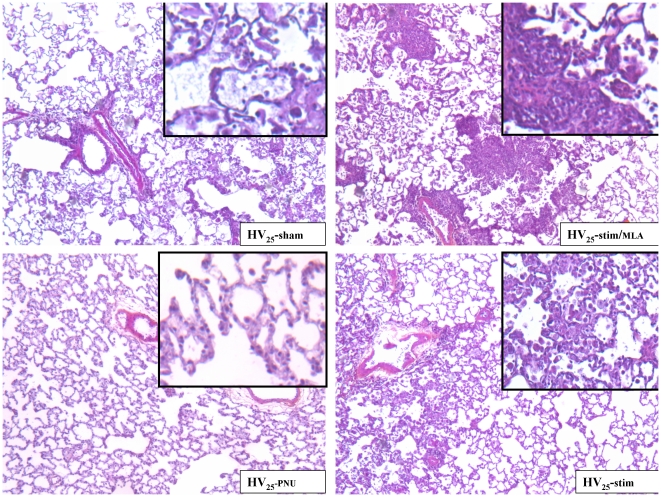
Lung histopathology of animals exposed to high stretch ventilation. Hematoxylin-eosin-saffron stained sections. Original magnification of pictures x50; original magnification of insets x100. The intra-alveolar inflammation with alveolar macrophages can be seen in the HV_25_-sham slide and, at a lesser level, in the HV_25_-stim slide. Note the alveolar polymorphonuclear leukocytes in addition to the macrophages in HV_25_-stim/MLA group. By contrast, no intra-alveolar inflammatory cell accumulation was seen in the HV_25_-PNU group. In every group, the inter-alveolar walls were moderately edematous and inflamed with inflammatory mononuclear cells. The alveolar edema is here especially marked on the HV_25_-sham slide.

Representative pathological slides are presented in figure 7.

**Figure 8:**
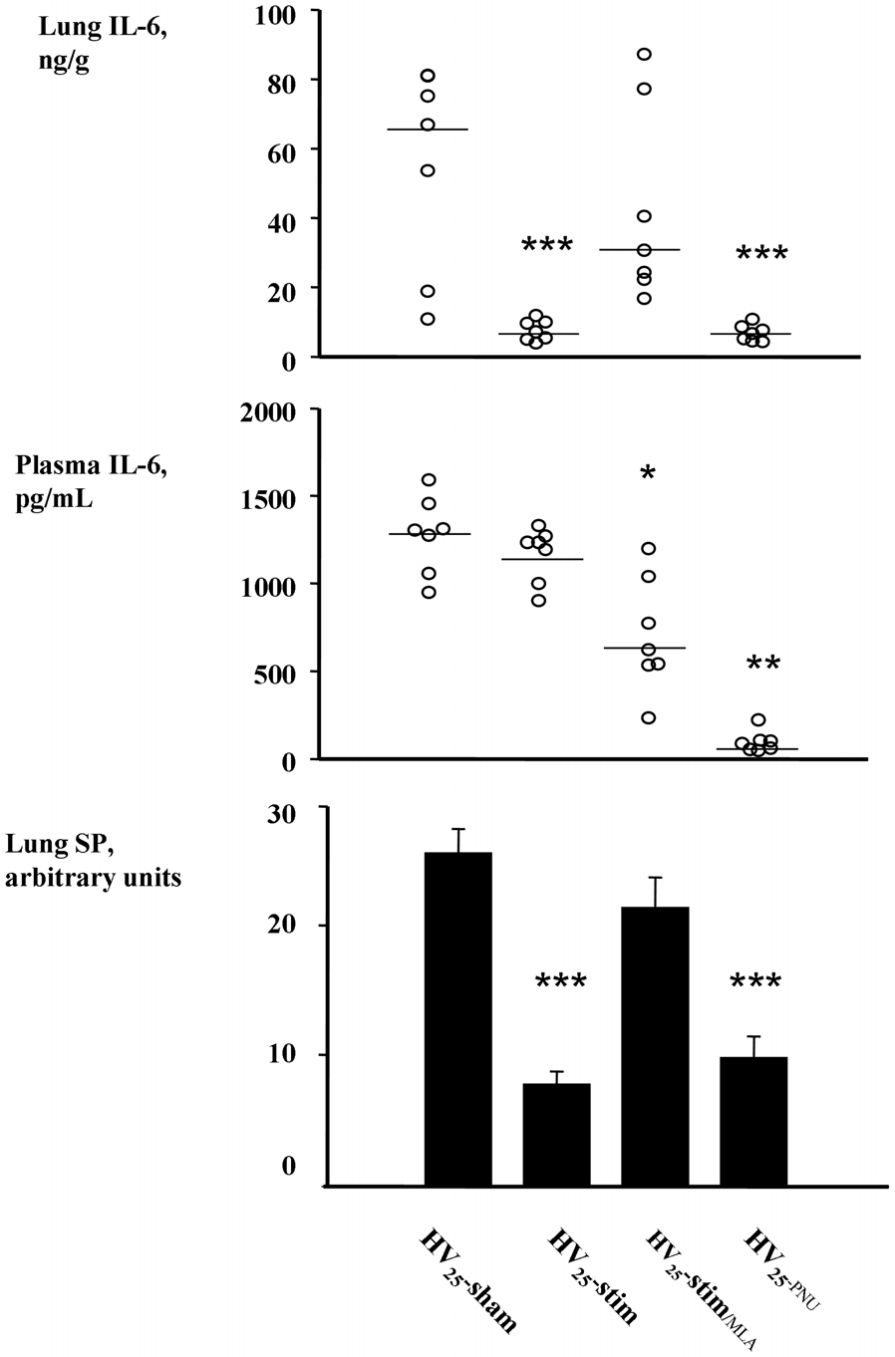
Concentrations of lung and plasma IL-6 and lung substance P contents after high pressure ventilation. IL-6 in lung extracts (top panel) and in plasma (middle panel), measured by ELISA. SP lung contents assessed by HPLC (bottom panel). Individual (circles) and median (horizontal bars) values are indicated for IL-6. SP is presented as mean values (solid bars) and standard error. **HV_25_-sham**: injurious ventilation. **HV_25_-stim**: injurious ventilation associated with vagal stimulation. **HV_25_-stim/MLA**: injurious ventilation associated with vagal stimulation and pre-treatment with the alpha7 nicotinic acetylcholine receptor blocking agent MLA. **HV_25_-PNU**: injurious ventilation associated with pre-treatment with the alpha7 nicotinic acetylcholine receptors agonist PNU. Asterisks indicate significant differences versus HV_25_-sham group (*p<0.05, ** p<0.01; *** p<0.001).

As shown in figure 8, both HV_25_-stim and HV_25_-PNU groups had a strong reduction in lung IL-6 concentration when compared to the HV_25_-sham group (median [25–75 percentiles], ng/g : 66.9 [25.6–79.5] in HV_25_-sham versus 2.2 [2.0–2.4] in controls, p<0.001). The SP lung content was also reduced (figure 8).

Plasma levels of IL-6 are presented in figure 8 panel B. Among the groups in which animals survived for 2 hours, the HV_25_-_PNU_ group was the only one to show a significant final decrease in IL-6 plasma level when compared to the HV_25_-sham group (median [25–75 percentiles], pg/mL: 1309.0 [1276.0–1456.0] versus 40.6 [36.3–45.0] in HV_25_-_PNU_, p<0.001). It must be noticed that in the HV_25_-stim/MLA group, the plasma was sampled earlier because of premature deaths (<60 min.) which explains lower final IL-6 amounts (bias due to the lower duration of exposition).

## Discussion

The present work confirms that high stretch ventilation applied for 2 hours induces lung injury and inflammation in rats. In this model, vagal electro-stimulation or pre-treatment with a selective agonist of the α7-nAChR, limited largely these consequences. Conversely, after blockade of the α7-nAChR, vagal stimulation failed to protect and lung damage was even more intense. These results confirm the existence of a vagally mediated reduction in VILI through the cholinergic nicotinic pathway. Besides, by using a uniformly designed experiment in rats, we originally demonstrate in vivo the preventive effect of a pre-treatment with a α7-nAChR agonist.

The involvement of the α7-nAchR has been recently demonstrated in vitro in a study showing reduction in cell stretch-induced inflammation and apoptosis [14]. We reinforced its findings by demonstrating that activation of the α7-nAchR by a specific agonist can prevent impairment in lung function and inflammation provoked by lung stretch in vivo. Surprisingly, the protection reported here with the α7-nAchR agonist was nearly complete, whereas it was only mitigated with a global pharmacological activation of the vagus nerve in mice [14]. Such a more intense effect could be due in part to our different animal model (rats), to the avoidance of hypocapnia, which is known to favor VILI [23], and to our shorter length of exposition to high stretch ventilation (two versus three hours). Although we didn’t demonstrate or study it here, the specific α7-nAchR activation with PNU-282987 was perhaps more powerful than with a global vagomimetic drug such as CNI-1493 [24]. One may however wonder about PNU-282987 efficiency over the study period since its half life has not yet been reported in rats following intraperitoneal injection. We think it unlikely that PNU-282987 had become fully inactive after two hours, because it has a high affinity for the α7 nicotinic acetylcholine receptors and its half life value in vitro is >120 minutes [25].

Since IL-6 concentrations were a hundred-fold higher in lung than in plasma, it is highly likely that IL-6 was mainly lung borne. The similar plasma concentrations of IL-6 observed in the HV_25_-sham and HV_25_-stim groups may result from a peripheral production in response to the cardio-vascular deterioration due to high thoracic pressures, with subsequent tissue hypoperfusion. The α7-nAChR agonist injected into the systemic circulation seemed able to decrease the IL-6 production in the whole organism, perhaps because of a better hemodynamics, or because of a down-regulation applied to the whole organism in opposition to the vagal stimulation which should have released acetylcholine into the thoraco-abdominal organs only.

Because there is a large spread of vagal innervation into the lung [26], acetylcholine can be released from efferent vagus nerves all along the respiratory system, from the trachea to the respiratory bronchioles. Some reports demonstrate that α7-nAChR are expressed by various non-neuronal cells [27], [28], especially lung macrophages and alveolar type II cells [3], these cells being thus the possible targets for acetylcholine binding. Some inhibitory signaling pathways have been experimentally highlighted suggesting how activation of the α7-nAChR may downregulate inflammation. For instance, in human monocytes stimulated with lipopolysaccharide, activation of the α7-nAChR by nicotine suppressed NF-κB nuclear translocation and I-κB phosphorylation, and, in mouse macrophages previously activated by intestinal manipulation, the acetylcholine released during vagus nerve stimulation activated the inflammation-inhibitory JAK2/STAT3 pathway. Both experimental situations resulted in downregulation of pro-inflammatory cytokines, including IL-6 [8], [29], [30]. In the present work, global vagal stimulation or specific pharmacological activation of the α7-nAChR minimized the adverse effects of high stretch ventilation and strongly decreased lung IL-6 and SP contents. IL-6 has been known to be downregulated by the cholinergic anti-inflammatory pathway since the princeps article of Borovikova et al. [6]. On the other hand, SP is a well known inflammatory mediator. Whether α7-nAChR directly interacts with SP would need specific studies and has not been assessed in the present work. A link has been shown between SP and cytokines in human blood monocytes, SP activating the production of cytokines, especially IL-6 [31]. Reciprocally, cytokines injected into the lung (especially IL-1 beta but possibly IL-6), can activate SP containing C-fibers [32], [33]. Therefore, the α7-nAChR activation could decrease indirectly the SP amounts through a lower cytokine release by the cells expressing this receptor.

The reduction in stretch-induced lung damage measurable both on the physiological and pathological levels could result partly from the inhibition of cytokine/SP release, but not only. As discussed above, the less pronounced fall in blood pressure of the animals receiving PNU may have favored less cytokine release (especially in plasma). In this group, a preservation of cardiovascular function, as suggested from blood pressure, heart rate, and pH data, may also have contributed to lower lung weight. On the other hand, the knowledge of biological and functional effects of α7-nAChR activation is only starting. Some reports suggest its involvement in systemic vascular tone [34] or pulmonary vascular permeability [3]. The possibility of a beneficial effect of α7-nAChR activation, for example on pulmonary perfusion, vascular permeability or cardio-vascular tone, cannot be discarded. In the HV_25_-stim group, some degree of lung injury remained: there was a larger range of lung weight values and a persistency of lung pathological abnormalities. Despite the fact that crude lung weight is not a very sensible index, it was corroborated by data on compliance and pathological findings. In the HV_25_-stim group, repeated periods of bradycardia and hypotension may have induced heart failure, which could have generated some degree of lung edema and only partial protection against inflammation. In this group, the cardiovascular consequences of vagal stimulation were probably underestimated because we recorded the heart rate and arterial pressure values once vagal stimulation had been stopped. This could explain the significantly lower pH value at 60 minutes in this group (metabolic acidosis). In addition, a specific inflammatory effect of vagal stimulation on airways could have contributed to the lung inflammation in this group [35] explaining the incomplete protection with regard to inflammation.

The idea of a strong protective role of the α7-nAChR was reinforced by the observation of a worsening in lung damage when vagal stimulation was preceded by α7-nAChR blockade with MLA. The reasons for such a worsening can only be speculative. We previously reported that the vagus nerve participates in lung SP release and injury during high stretch ventilation [22]. The stimulation of the whole vagus nerve releases SP retrogradely from afferent C-fibers [36], [37] and, orthodromically, acetylcholine from efferent fibers [26]. It is possible that, in the HV_25_-stim group, the cholinergic anti-inflammatory pathway had downregulated the stretch-induced inflammatory response since the α7-nAChR were reachable. With α7-nAChR blockade coupled to vagal stimulation, a possible vagally-mediated SP increase may have increased inflammation [38]. Because lung SP amounts may result both from neurogenic (vagal) and non-neurogenic (immune cells) sources, it is not surprising that lung SP was reduced in the HV_25_-stim group given the anti-inflammatory effect of the cholinergic pathway. MLA can also inhibit a population of non- α7-nAChR such as α4β2-nAChR [39], which has also been involved in anti-inflammatory processes [40]. The fact that the enhanced injury observed in the HV_25_-stim /MLA group could result partly from the blockade of these other receptors should therefore not be discarded. Another possible explanation for worsening in the HV_25_-stim/MLA group is based on basic physiological knowledge: heart activity depends on the balance between the inhibitory effects of the parasympathetic innervation and the stimulatory effects of the sympathetic innervation. The α7-nAChR also mediates autonomic ganglionic transmission [41] and its blockade can prevent increase in sympathetic cardio-vascular activity [18]. Therefore, in our experiment, the reported premature deaths with cardio-circulatory failure in the HV_25_-stim/MLA group could result from the inability of the sympathetic system to counterbalance the vagally-induced inotropic and bathmotropic negative effects, as suggested by the bradycardia reported in figure 5. The half life of MLA being about 20 minutes in rats after intravenous injection [42], we think it likely that efficient concentrations of this drug were still present in the plasma of the HV_25_-stim/MLA group at the time of death, occurring at twice the half life.

The present study confirms the previously suggested modulation of lung inflammation by the cholinergic anti-inflammatory pathway in the lungs exposed to VILI. Selective pharmacological stimulation of the α7-nAChR appears efficient in preventing VILI and might be a new pharmacological target, especially in ARDS patients at risk for ventilator associated injury. However, further experiments with longer duration of mechanical ventilation and applied to larger animal models would be required to confirm the benefits observed.

## Supporting Information

Figure S1 **Comparison between HV_25_-sham animals (large volume ventilation generating 25 cmH_2_O airway pressure and saline injection) and HV_25_-MLA (large volume ventilation generating 25 cmH_2_O airway pressure and pre-treatment with alpha7 nicotinic acetylcholine receptor blocking agent MLA).** Data are expressed as mean ± SEM. Panel A: time course of systolic arterial blood pressure (bars), and of airway pressure (line and plots), in animals exposed to high stretch ventilation. Paw: airway pressure. The changes over the time were significant versus baseline in both groups but no significant difference between the two groups was detected. Panel B: pressure-volume relationships. Paw: airway pressure. Panel C: weight of the right lung indexed to the rat body weight as measured at the end of the injurious ventilation period; ns : no significant difference. Panel D: compliance of the respiratory system measured at inflation (Crsi) indexed to the rat body weight (Crsi/BW); ns : no significant difference.
(TIF)

Figure S2 **Example of the response to the bilateral stimulation of the cervical vagus nerves during high stretch ventilation.** Upper and lower tracings represent respectively the airway pressure (Paw) and the arterial blood pressure (ABP). The bold horizontal arrow represents the beginning of a 10-min period of electrical stimulation. Peripheral vagal stimulation induced bradycardia and hypotension attesting its remaining functional efficacy, in this case, 90 min after the start of the experiment. It must be noted that cardiovascular changes partly adapted when vagal stimulation was continued (parasympathetic overdrive). The Paw remained at 25 cmH_2_O during the vagus nerve stimulation.
(TIF)

Figure S3 **Example of the responses to the bilateral stimulation of the cervical vagus nerves before injurious ventilation, i. e. during the conventional ventilation applied for the first ten minutes of the experiment in one HV_25_-stim rat.** Upper and lower tracings represent respectively the airway pressure (Paw) and the arterial blood pressure (ABP). The bold horizontal line represents the electrical stimulation. The recording was performed just after the injection of the neuromuscular blocking agent and some residual spontaneous inspirations generated negative Paw waves. In parallel to the hypotention, an increase in airway pressure (Paw) can be observed, attesting of the vagally-induced bronchospasm.
(TIF)
