# Supplementary figures and images for: Activation of Nicotinic Cholinergic Receptors Prevents Ventilator-Induced Lung Injury in Rats
Source: PLoS One. 2011 Aug 8;6(8):e22386. doi: 10.1371/journal.pone.0022386 (PMC3152549; doi:10.1371/journal.pone.0022386)

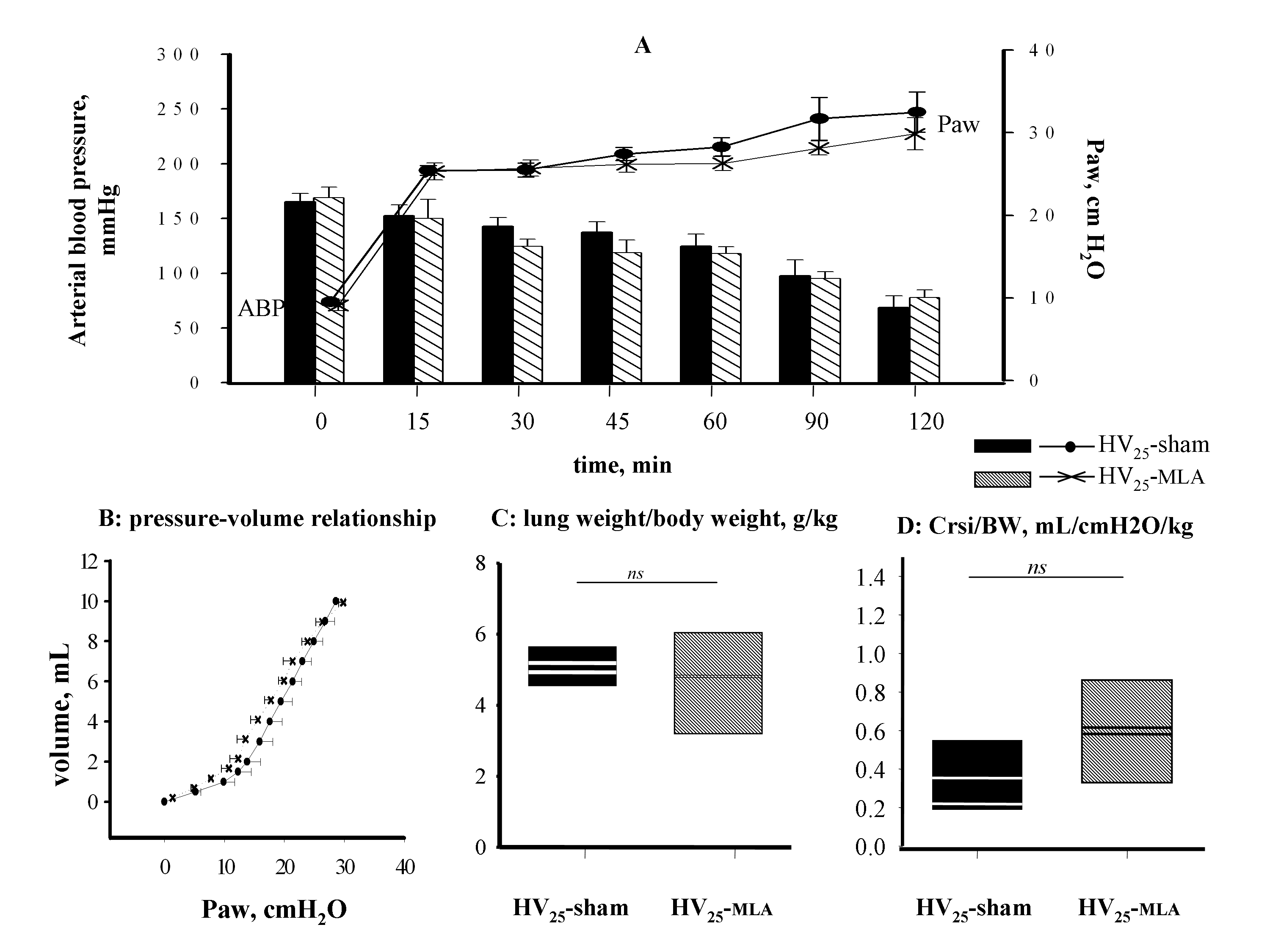

Supplement: Figure S1 — Comparison between HV25-sham animals (large volume ventilation generating 25 cmH2O airway pressure and saline injection) and HV25-MLA (large volume ventilation generating 25 cmH2O airway pressure and pre-treatment with alpha7 nicotinic acetylcholine receptor blocking agent MLA). Data are expressed as mean ± SEM. Panel A: time course of systolic arterial blood pressure (bars), and of airway pressure (line and plots), in animals exposed to high stretch ventilation. Paw: airway pressure. The changes over the time were significant versus baseline in both groups but no significant difference between the two groups was detected. Panel B: pressure-volume relationships. Paw: airway pressure. Panel C: weight of the right lung indexed to the rat body weight as measured at the end of the injurious ventilation period; ns : no significant difference. Panel D: compliance of the respiratory system measured at inflation (Crsi) indexed to the rat body weight (Crsi/BW); ns : no significant difference. (TIF) [file pone.0022386.s001.tif]

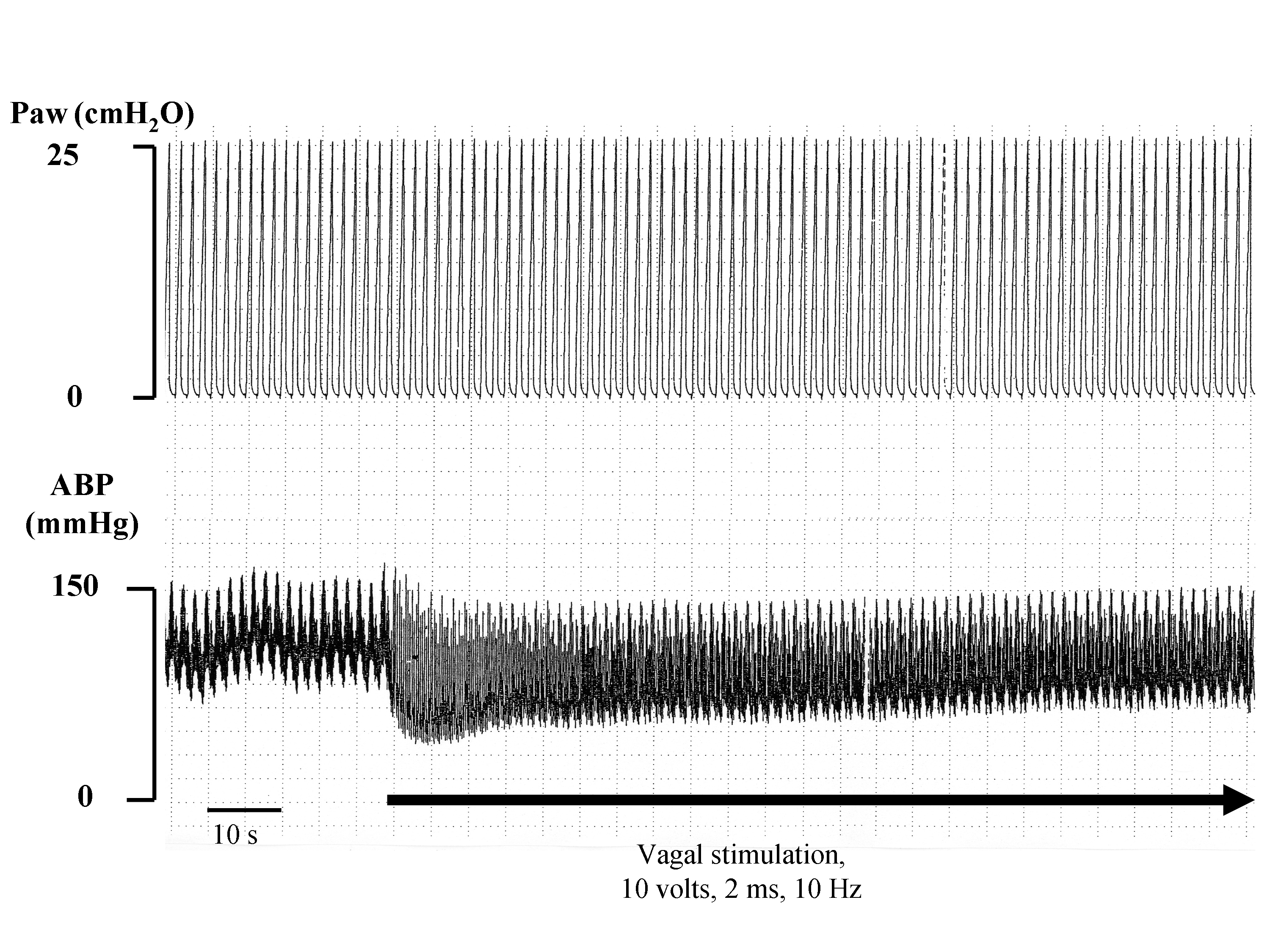

Supplement: Figure S2 — Example of the response to the bilateral stimulation of the cervical vagus nerves during high stretch ventilation. Upper and lower tracings represent respectively the airway pressure (Paw) and the arterial blood pressure (ABP). The bold horizontal arrow represents the beginning of a 10-min period of electrical stimulation. Peripheral vagal stimulation induced bradycardia and hypotension attesting its remaining functional efficacy, in this case, 90 min after the start of the experiment. It must be noted that cardiovascular changes partly adapted when vagal stimulation was continued (parasympathetic overdrive). The Paw remained at 25 cmH2O during the vagus nerve stimulation. (TIF) [file pone.0022386.s002.tif]

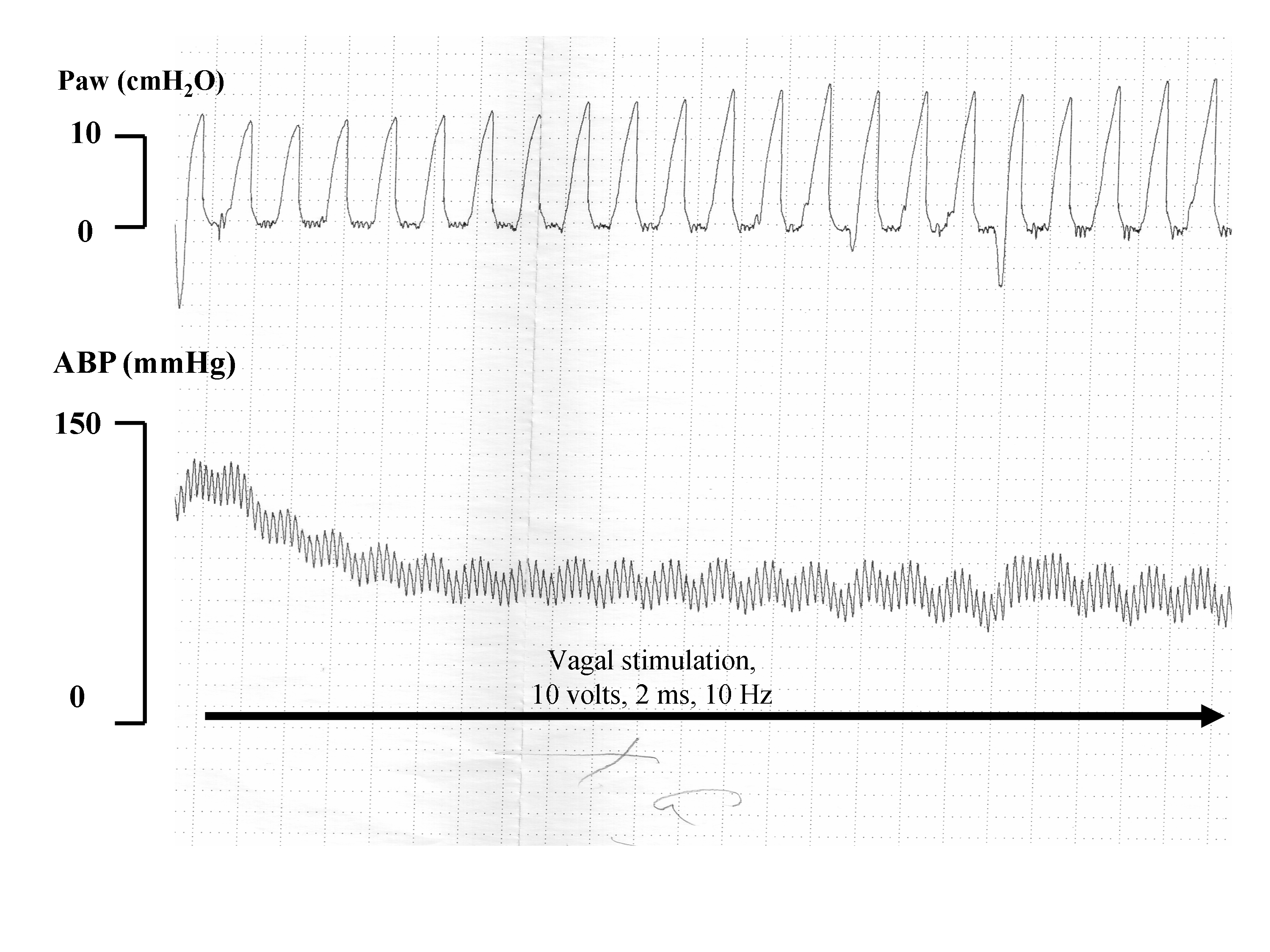

Supplement: Figure S3 — Example of the responses to the bilateral stimulation of the cervical vagus nerves before injurious ventilation, i. e. during the conventional ventilation applied for the first ten minutes of the experiment in one HV25-stim rat. Upper and lower tracings represent respectively the airway pressure (Paw) and the arterial blood pressure (ABP). The bold horizontal line represents the electrical stimulation. The recording was performed just after the injection of the neuromuscular blocking agent and some residual spontaneous inspirations generated negative Paw waves. In parallel to the hypotention, an increase in airway pressure (Paw) can be observed, attesting of the vagally-induced bronchospasm. (TIF) [file pone.0022386.s003.tif]
